# Discovering and understanding oncogenic gene fusions through data intensive computational approaches

**DOI:** 10.1093/nar/gkw282

**Published:** 2016-04-21

**Authors:** Natasha S. Latysheva, M. Madan Babu

**Affiliations:** MRC Laboratory of Molecular Biology, Francis Crick Ave, Cambridge CB2 0QH, United Kingdom

## Abstract

Although gene fusions have been recognized as important drivers of cancer for decades, our understanding of the prevalence and function of gene fusions has been revolutionized by the rise of next-generation sequencing, advances in bioinformatics theory and an increasing capacity for large-scale computational biology. The computational work on gene fusions has been vastly diverse, and the present state of the literature is fragmented. It will be fruitful to merge three camps of gene fusion bioinformatics that appear to rarely cross over: (i) data-intensive computational work characterizing the molecular biology of gene fusions; (ii) development research on fusion detection tools, candidate fusion prioritization algorithms and dedicated fusion databases and (iii) clinical research that seeks to either therapeutically target fusion transcripts and proteins or leverages advances in detection tools to perform large-scale surveys of gene fusion landscapes in specific cancer types. In this review, we unify these different—yet highly complementary and symbiotic—approaches with the view that increased synergy will catalyze advancements in gene fusion identification, characterization and significance evaluation.

## INTRODUCTION

**Figure 1.**
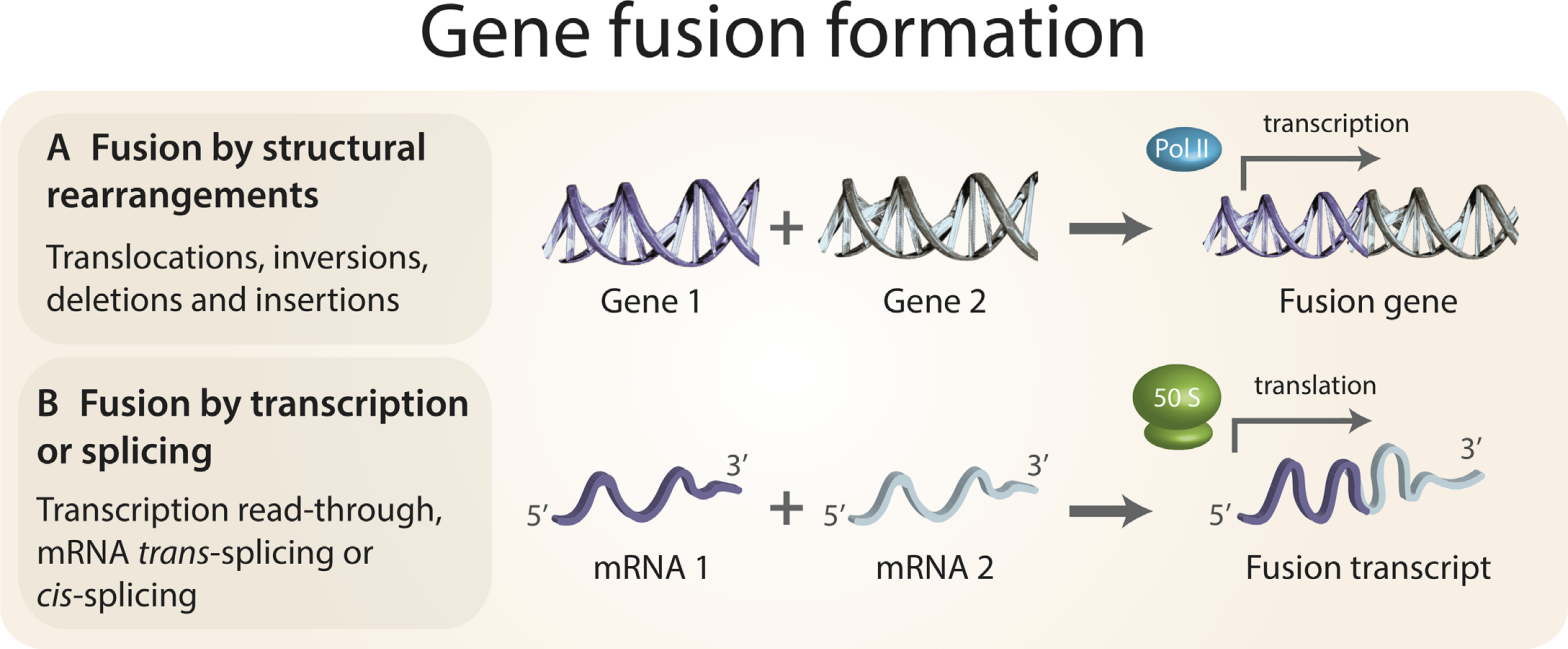
Mechanisms of gene fusion formation. (**A**) Structural rearrangements of chromosomes, such as translocations, inversions, deletions and insertions, can lead to the formation of gene fusions. These hybrid genes may then be transcribed and translated into fusion transcripts and proteins. (**B**) Non-structural rearrangement mechanisms, such as transcription read-through of neighboring genes or splicing of mRNA molecules, are increasingly recognized as leading to the formation of a large proportion of gene fusions.

Gene fusions are hybrid genes formed when two previously independent genes become juxtaposed. The fusion can result from structural rearrangements like translocations and deletions, transcription read-through of neighboring genes (1–3), or the *trans*- and *cis*-splicing of pre-mRNAs (4–8) (Figure 1). Many gene fusions are associated with oncogenic properties, and often act as driver mutations in a wide array of cancer types (9,10). Gene fusions commonly exert their oncogenic influence by either deregulating one of the involved genes (e.g. by fusing a strong promoter to a proto-oncogene), forming a fusion protein with oncogenic functionality (e.g. by causing a constitutive activation of a tyrosine kinase) or inducing a loss of function (e.g. by truncating a tumor suppressor gene). One estimate states that translocations and gene fusions are responsible for 20% of global cancer morbidity (11), largely due to their central involvement in prostate cancer. Recent bioinformatics advances have elucidated many aspects of oncogenic gene fusions, from the origin and causative importance of fusion events, to the structural and regulatory properties of fusion proteins.

The total number of gene fusions is now estimated to be 10 000, with over 90% of these being identified in the past 5 years due to advances in deep-sequencing and fusion detection algorithms (12). The prevalence of gene fusions varies widely between cancer types (10): at one extreme, gene fusions occur in (and frequently drive) 90% of all lymphomas, over half of leukemias (13), and one third of soft tissue tumors (14). In prostate cancer, one specific fusion (*TMPRSS2-ERG*) is the most common genetic alteration, being found in over 50% of patients (15). However, many recurrent gene fusions occur at low frequencies, such as the *KIF5B-RET* fusion, which is present in 1–2% of lung adenocarcinomas (16).

Knowledge of both common and rare gene fusions has improved numerous aspects of clinical care. For example, the *TMPRSS2-ERG* fusion transcript functions as a urinary biomarker for prostate cancer risk and prognosis (17) and gene fusions are used in the diagnosis of a variety of cancers (14,18,19). Gene fusions have also been important in identifying molecular subtypes of cancers (19–21), patient stratification (22,23), monitoring residual disease post-treatment (24,25) and predicting relapse (25). Importantly, fusion transcripts are also promising therapeutic targets (19,26–28). As an example, the development of drugs that target the ATP-binding sites (29) and allosteric regions (30) of the *BCR-ABL* fusion kinase, a constitutively active tyrosine kinase and the driving mutation in chronic myelogenous leukemia, has significantly improved patient outcome. Similarly, inhibitors of the anaplastic lymphoma kinase (ALK) protein have greatly improved prospects for patients with *EML4-ALK* fusion positive non-small cell lung tumors (31).

Although fusions have been recognized drivers of cancer for over 30 years, recent bioinformatics studies have substantially enriched our knowledge of fusions. However, the computational gene fusion literature is dispersed—for example, many fusion landscape studies make little reference to bioinformatics surveys of gene fusion molecular biology, which could help elucidate the function of novel fusions and set them into the context of other known oncogenic fusions. Similarly, an increased awareness of fusion prioritization algorithms could aid investigators in narrowing down putative fusion lists to only the instances that are likely to be biologically functional. This review aims to promote increased exposure and collaboration between different gene fusion researchers, especially those involved in identifying and describing novel fusions. In **Section 1**, we discuss the findings of recent data-intensive computational methods to study global properties of gene fusions, including gene fusion landscapes across different cancer types and the structural and regulatory characteristics of fusion proteins. In **Section 2**, we briefly outline fusion detection tools before focusing on reviewing computational approaches for prioritizing driver fusions and efforts to catalog and annotate oncogenic gene fusions within specialized databases.

## DATA-INTENSIVE COMPUTATIONAL STUDIES OF GENE FUSION FUNCTIONALITY

Bioinformatics approaches have been crucial to identifying global patterns in gene fusion functionality. In this section, we outline the recent computational work on the molecular functions, structural design principles and regulatory features of fusion proteins across diverse cancers.

### Global trends in gene fusion formation and function

**Figure 2.**
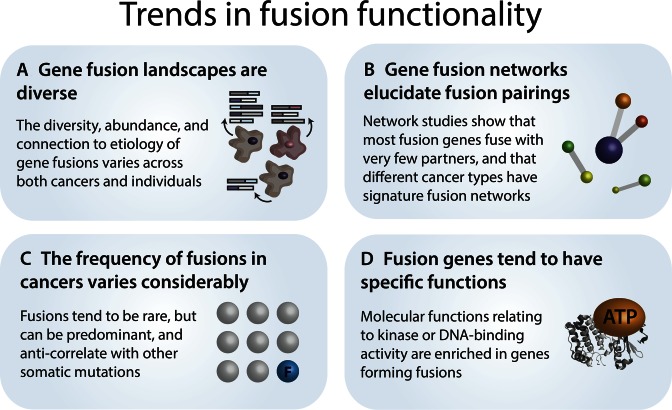
Trends in fusion functionality. (**A**) Recent surveys have uncovered the diverse gene fusion landscapes present in a variety of cancers. (**B**) The frequency of gene fusions varies by cancer type and appears to anti-correlate with frequencies of other somatic mutations at the level of both cancer types and individual tumor samples. (**C**) Gene fusions tend to involve genes with kinase, DNA-binding and chromatin modifying activity. (**D**) Network studies of fusions have identified global and cancer-type-specific patterns in gene partnerships, such as the trend toward most fusion genes only fusing with only one other partner.

Gene fusion landscapes have now been studied in many cancer types, including breast (32–34), lung (35), prostate (36–39), lymphoid (40), soft tissue (14) and gastric cancer (3) (see (19) for a collection of fusion landscape studies in epithelial cancers). Such studies have generated diverse insights, such as the finding that gene fusions are the major genomic abnormality in glioblastoma multiforme (41) and the discovery that private gene fusions cause an aggressive type of prostate cancer (42). The biology of certain rare cancers has been elucidated by the discovery of frequent oncogenic fusions, including the *C11orf95-RELA* fusion in supratentorial ependymoma (43) and the recurrent *DNAJB1-PRKACA* fusion in fibrolamellar hepatocellular carcinoma (44). These large-scale surveys continue to underscore the importance of screening for gene fusions (Figure 2A).

Given the expanding list of known gene fusions in cancer, it is important to understand the types of genes that frequently form fusions and what partners they fuse with. Gene fusion networks, in which nodes are individual genes and edges indicate the occurrence of a fusion between those genes, offer an organized approach to studying fusion partnerships (Figure 2B). Several studies of gene fusion networks have found that the majority of fusion genes partner with a single other gene, with only a few genes being highly promiscuous (11,12,45–47). An extreme example of promiscuity is the mixed lineage leukemia (*MLL*) gene, which fuses with over 60 different partner genes, and causes most infant leukemias and a significant proportion of adult leukemias (48). The set of fusion partners for a given gene may be influenced by the position of those partners in protein interaction networks (49), their domain content (46) or their structural capabilities (e.g. oligomerization ability in *FGFR* fusion partners (50)), but these concepts require further investigation. Höglund *et al*. performed the pioneering work on gene fusion networks using 291 oncogenic gene fusions from the Mitelman database (45). In addition to demonstrating that most fusion genes form few fusions, the fusion network was found to be fragmented-fusion pairs from hematological, mesenchymal and epithelial tumors tended to localize to different sections of the network, suggesting that gene fusion pairs are segregated according to tumor histology. However, this type of fragmentation in gene fusion networks may have been due to incomplete knowledge—in an updated gene fusion network analysis with 358 gene fusion pairs (11), 89% of genes formed three large interconnected networks, compared to 72% in the previous study. This updated gene fusion network study confirmed both the presence of several highly promiscuous fusion genes (e.g. *MLL, ETV6, EWSR1*) and many poorly connected ones, and also the apparent grouping of the network by cancer type (11). Interestingly, gene fusion networks can differ substantially in their topology across different cancer types—for example, the gene fusion network in acute myelogenous leukemia is clustered around a few genes (like *MLL* and *NUP98*), whereas the ovarian cancer gene fusion network is much more dispersed, with very few genes fusing with more than one partner (12). The basis for these cancer subtype-specific differences in gene fusion networks remains to be explained.

Several bioinformatics studies have searched for trends in fusion frequencies across cancers (Figure 2C). In general, the frequency of recurrent fusion transcripts is much lower than other somatic mutations (51). For example, in lung adenocarcinoma, the *EML4-ALK* driver fusion occurs at a rate of 6%, while driver mutations in *KRAS* and *EGFR* are much more common (rates of 25 and 23%, respectively) (52). Furthermore, the rates of gene fusions vary significantly across cancer types (11). A recent bioinformatics survey of gene fusions in TCGA identified 7887 high confidence fusion transcripts, with substantial differences in fusion frequencies across tumor types (highest rates in bladder cancers and the lowest in thyroid carcinoma) (10). Interestingly, the same study discovered a significant anti-correlation between frequencies of recurrent in-frame fusion transcripts and other gene mutations, hinting at potential oncogenic redundancy. However, given that in most tumors, >80% of fusion transcripts were associated with genomic instability (DNA amplification or deletion), it is unclear what proportion of gene fusions are oncogenic drivers rather than instability-induced passengers. The association between genomic instability and gene fusions has been previously reported (12,53), and it has been suggested that non-recurrent, singleton gene fusions are potential passenger mutations (54).

One open question concerns whether gene fusions play similar roles in different cancer types (Figure 2D). There are some hints that gene fusions in carcinomas are more likely to disrupt cell signaling processes involved in cell proliferation and homeostasis (55) than in hematopoietic and mesenchymal cancers, possibly due to differences in differentiation history. However, the same broad categories of genes tend to be fused in all cancers—predominantly, kinases and transcriptional control genes (56). Fusions involving kinases have been extensively documented as an important class of gene fusions (54,57) and are especially interesting due to their susceptibility to kinase inhibitors (58). Recently, at least one in-frame kinase fusion was found in 7.4% of analysed tumor samples in TCGA, with the highest rates occurring in thyroid carcinoma. This suggests that kinase inhibition will continue to grow as a promising treatment option for kinase fusion-positive cancers (10). Another study of kinase fusions also reported the highest rate of recurrent kinase fusions in thyroid cancers, and further found that fusions between the kinases *ALK, BRAF, MET, NTRK1, NTRK2, RAF1* and *RET* were mutually exclusive (54). Histone methyltransferases are increasingly recognized as another attractive drug target in cancer (59), and were found to be fused in-frame in 2.5% of all tumor samples in TCGA in a mutually exclusive manner with protein kinase fusions (10). Finding the rationale behind observed patterns of mutual exclusivity between gene fusions and other oncogenic mutations, as well as between different types of gene fusions, is likely to be a fruitful avenue for future research.

Many gene fusions are found across a variety of different cancer types. For example, *RAF* kinase family fusions have recently been profiled across a dozen different solid tumors (60), and FGFR tyrosine kinase fusion proteins (which interestingly, occasionally exclude the tyrosine kinase domain) are similarly promiscuous (50). In such cases, treatments developed for a specific cancer type can potentially be used to treat another. For instance, FGFR fusion proteins have emerged as promising therapeutic targets across the spectrum of cancers they are detected in (27,50,61–64). A growing number of studies seek to target oncogenic fusion transcripts and proteins, and an overview of recent therapeutic work has recently been written (19), together with a compiled list of 33 recent clinical trials targeting epithelial cancer fusions (Additional File 2 within reference). Fusions involving the *ALK, ETS* and *RET* genes dominate this list (19), suggesting that updates to the treatment repertoires of the cancers affected by these fusions are forthcoming.

Knowledge of how precisely fusion transcripts or proteins have been successfully targeted will be valuable for future drug development studies. One important success story is the treatment of non-small-cell lung cancer with ALK inhibitors (65). The transforming *EML4-ALK* fusion transcript was initially detected in approximately 7% of patients with non-small-cell lung cancer (NSCLC) (66), with the fusion being mutually exclusive with the better-known *EGFR* mutations. The EML4-ALK fusion protein consists of the N-terminus of the microtubule-associated EML4 protein and the C-terminus of the ALK receptor tyrosine kinase, which itself contains an intact tyrosine kinase domain that mediates ALK oligomerization and the subsequent induction of constitutive kinase activity. Similarly to many other fusions, the tyrosine kinase domain of ALK was from the start found to be core to the oncogenic activity of the EML4*-*ALK fusion protein (66). Highly effective and well-tolerated ALK inhibitors, such as crizotinib, were rapidly developed for therapeutic purposes (65,67–69). Crizotinib inhibits the ALK tyrosine kinase by binding to its ATP-binding pocket, and the introduction of this drug substantially improved prospects in both *EML4-ALK* positive (31,65) and *ROS1* fusion positive NSCLC patients (70,71). However, through a variety of mechanisms (72–76), both *ALK* and *ROS1* fusion positive NSCLC are susceptible to developing resistance to crizotinib, and current research focuses on overcoming this resistance (77–79). The prevalence of gene fusions involving kinases (54) such as *ALK* (80,81), together with the relatively high success of targeting kinases in cancer (82), suggest that research focusing on inhibiting deregulated fusion kinases will continue to pay dividends. Notably, nuanced knowledge of the specific structural variants of the same type of fusion protein (33) and the downstream signaling of fusion proteins (83) can be key to developing successful therapeutic agents, reinforcing the need for larger scale computational studies which can detect these molecular trends and suggest candidate targets.

### Structural properties of fusion proteins

Fusion transcripts can be translated into fusion proteins, though estimates of translation frequency vary (84). Predicting the function of fusion products is non-trivial, but is distinct from the extremely difficult task of predicting a protein's function from a sequence. One can attempt to infer a fusion protein's function by examining the structural and regulatory features of the parent proteins it is composed of, as well as the precise segments of the parent proteins that form the fusion product. A variety of studies have tried to understand the underlying structural design principles of fusion proteins by examining translocation breakpoint positions, domain architectures and the role of intrinsic structural disorder.

**Figure 3.**
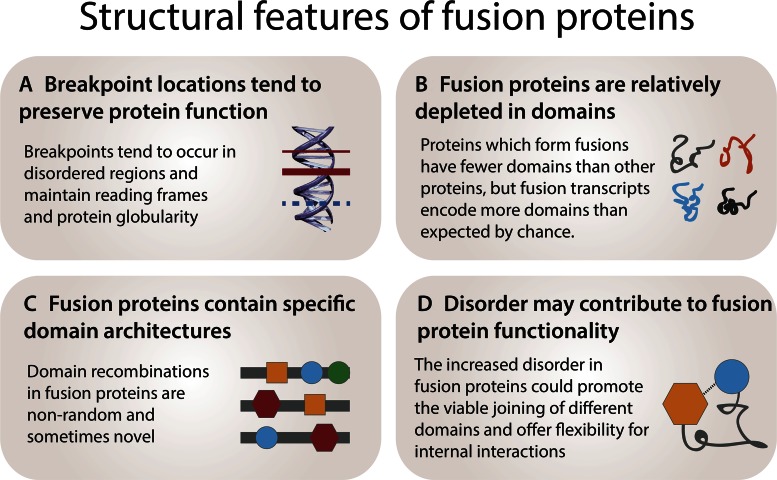
Structural features of fusion proteins. (**A**) Genes which form fusions tend to have fewer domains, but fusion transcript sequences have been shown to have more domains than expected by chance. (**B**) Fusion proteins are enriched for specific domains and permutations, which are occasionally proteomically novel. (**C**) Fusion breakpoints are biased toward locations which preserve fusion protein reading frames and structural viability. **(D)** Increased intrinsic disorder in fusion proteins may permit the protein to fold and place the constituent domains into proximity of each other.

The location of translocation breakpoints in cancer is known to be non-random and recurrent, and has been extensively demonstrated to be influenced by both the spatial proximity of chromosomes in the nucleus as well as features of the DNA sequence, such as repeats, fragile sites and endonuclease misrecognition sites (85) (Figure 3A). However, the structure and function of the resulting fusion proteins has emerged as an additional force governing breakpoint locations. For example, one global analysis of fusion transcripts reported that translocation breakpoint positions almost universally (98%) conserve reading-frame compatibility (46). However, the most recent census of fusions across 13 tumor types reported that 36% of detected fusion transcripts are in frame, with AML and thyroid carcinoma having the highest rates of reading frame conservation (79 and 70%, respectively) (10). Interestingly, these were also the cancer types with the highest rates of balanced translocations. More recently, a study aimed at discovering novel gene fusions in prostate cancer found a very limited number of feasible transcripts, and most were not in frame (36). A fusion prioritization study found that in-frame transcripts were the most powerful predictor of driver fusions (86), confirming the intuition that in-frame transcripts are crucial to function. These conflicting reports appear to suggest that rates of reading frame conservation—which is likely to correlate with rates of functional and potentially driving gene fusions—may vary significantly across cancer types and samples.

Translocation breakpoints have been found to generally occur in intrinsically disordered regions, which may reflect a selection for regions that can more seamlessly combine different segments (87) (Figure 3A). Notably, breakpoints were also observed to preferentially avoid splitting domains, and in instances where globular domains were split, the truncations tended to generate viable proteins due to the breakpoints being positioned in low hydrophobicity regions. Together with frame-shift conservation (where it occurs), such trends could reflect a selection on fusion proteins to maintain viability and evade degradation pathways.

A study of intrinsic disorder in fusion proteins found that translocated proteins are more intrinsically disordered and tend to have fewer Pfam domains than non-translocated proteins (87) (Figure 3B), which has recently been demonstrated again (88). However, another survey of fusion proteins showed that they contain complete protein domains much more frequently than would be expected if fusion transcripts were generated from randomly fused protein coding sequences (89). Hence, domains may be relatively rare in fusion proteins but occur more often than random. Where fully functional domains are present in fusion proteins, these could in some cases compete with original proteins and produce dominant negative effects—for example, in their fusion protein set (89), DNA binding domains were found to be frequent but transcriptional activation domains were rare, which reflects a known dominant negative mechanism employed by some oncogenic fusion proteins (90). In general, fusions involving transcriptional activators or repressors could be liable to exerting dominant negative effects (89), and one of the best studied examples of this mechanism is the RUNX1-ETO fusion protein, which is implicated in the development of acute myeloid leukemia (91–93). RUNX1-ETO exerts a dominant negative influence over RUNX1, a crucial regulator of hematopoietic stem cell differentiation, by interfering with normal RUNX1 function and blocking differentiation. The fusion proteins typically retain the DNA-binding Runt homology domain from the RUNX1 transcription factor, thus inheriting the ability to bind to RUNX1 target genes, as well as incorporating most of the transcriptional repressor ETO protein, thereby allowing the fusion protein to act as a constitutive transcriptional repressor through several mechanisms. The result is the transcriptional repression of RUNX1 target genes, which is strongly implicated in leukemogenesis (91).

The types of domain combinations observed in fusion proteins have been relatively well studied (Figure 3C). A survey of fusion protein domain architectures demonstrated that the same architectures are reused in different gene fusion events, providing an underlying principle behind fusion networks (46). The most commonly reused architectures in fusion proteins involve tyrosine kinases, EWS activation domains and Runt domains. In general, domain combinations with closer links to oncogenic behavior are more frequently found (46). Another study compared Pfam domain permutations in 7424 fusion mRNAs to domains in known human proteins (89) and reported that although most domain types (69%) appear in fusion proteins, eight domain types are over-represented. These included AT hooks (involved in transcriptional regulation) and MHC and receptor tyrosine kinase catalytic domains (which are membrane protein and receptor domains). Interestingly, some fusion proteins encode novel combinations of domains not found in normal proteins, including pairings between DNA-binding HLH (helix-loop-helix) and GTP-binding domains as well as fusions between PHD-zinc finger and coiled-coil (DNA binding) domains (89). However, novel domain recombinations may be rare—fusions have been shown to preferentially include partners that, when fused, reconstitute known domain co-occurrences (49). Finally, a study of fusion protein exon and domain organization showed an enrichment of transmembrane domains and signal peptides in fusion proteins (84), which suggests that fusion protein functionality could be modulated by changing the cellular localization or context of biochemical functions.

In line with these findings, the presence of certain domains in fusion proteins has been shown to be predictive of driver fusions: the developers of the ConSig algorithm for fusion driver prioritization found that although domain architectures of fusion proteins were highly diverse, especially for 5′ partners, certain architectures were predictive of driver fusions (49). Interestingly, domain architectures did not appear to be significantly shared by sets of fusion partners of a given gene—that is, there was no evidence that recombination patterns of specific fusion partners were especially impacted by domain content. Furthermore, there was no apparent association between specific domain architectures and tumor types. However, other reports have found evidence for different domain patterns in partner sets and across cancer types (11,46,47). Further work is required to reconcile the apparent conflict (which may be due to differences in datasets) and develop a molecular model for observed fusion partnerships.

Gene fusions are formed from two partner genes, and these partners need not necessarily encode similar structural elements. A computational study of domains and protein–protein interaction (PPI) interfaces in fusion proteins found substantial differences in the structural properties of 5′ and 3′ fusion partner genes (47). Although both DNA-binding and PPI domains were most common in both 5′ and 3′ partners, kinase and histone modification domains were almost entirely absent in 3′ partners. The co-occurrence of domains in 5′ and 3′ partners is strongly correlated—for example, protein interaction domains disproportionately co-occur with DNA-binding and kinase domains, which is a combination that could conceivably lead to signaling defects (94). The retention patterns also differ between 5′ and 3′ partners: the 3′ partners tended to retain a significant portion of their domains and protein interaction interfaces, whereas the 5′ partners tended to lose domains, often retained no domains and in the instances where they did retain domains, these tended to lack a clear oncogenic function. The Oncofuse predictor for prioritizing driver fusions found that lost interaction interfaces were actually more predictive of drivers than retained ones, hinting at the importance of loss of parental protein function effects, in addition to gain of function effects (47,95). Still, the protein interactions of fusion proteins are likely to contribute to oncogenicity, as suggested by the observation that, in known fusion partners, there is a significant over-representation of domain–domain interactions among their constituent domains (49). These initial observations, as well as the recent successes in studying cancer mutations from the point of view of interaction networks (96–102), call for deeper analyses of fusion–protein interactions.

In addition to structured protein regions like domains, intrinsically disordered regions have been increasingly recognized as important functional players in the proteome and in disease (103,104) (Figure 3D). An early computational study found that translocated proteins are over twice as disordered as other human proteins, and this disorder may help mediate oncogenic functions by providing the flexibility necessary to allow the different elements in fusion proteins to synergistically interact (87).

Long non-coding RNAs (lncRNAs), which do not encode for proteins, have recently been the subject of interest in cancer research (105–107). However, possibly due to the fact that many fusion-detection pipelines filter out fusion candidates that do not map to protein-coding regions, only a handful of gene fusions involving lncRNAs have been documented. The list includes a fusion between *ETV1* and a prostate-specific lncRNA in prostate cancer (108,109) and the fusion of the *BCL6* proto-oncogene with the non-coding *GAS5* gene in a B-cell lymphoma patient (110). These lncRNAs may simply contribute to the aberrant regulation of their oncogene partner, rather than having an oncogenic function themselves (111). Additionally, a study of prostate cancer in Asian populations found several novel fusions involving lncRNAs (112), including a surprisingly common gene fusion between the *USP9Y* protease and the *TTTY15* ncRNA, which results in a fusion transcript and is associated with a loss of *USP9Y* function. This fusion has since been found to be an effective urine-based biomarker that is predictive of prostate biopsy outcomes (113). These few cases highlight the fact that fusions involving lncRNAs can be functional and even clinically relevant, and therefore the current approach of filtering out non-coding gene fusions may be systematically omitting substantial useful information.

To conclude, the previous bioinformatic studies of the structural aspects of fusion proteins suggest the following trends: fusion proteins are structurally diverse, but tend to be disordered and depleted in domains. However, certain domain combinations are enriched, such as those involving kinase and DNA-binding activity. Breakpoints tend to preserve in-frame translation and globularity, and 5′ and 3′ fusion partners generally contribute to different structural elements to fusion proteins.

### Expression and regulation of fusion proteins

**Figure 4.**
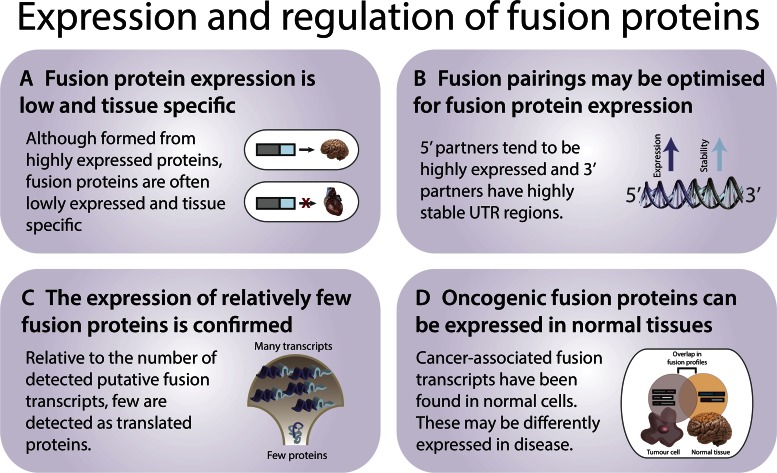
Expression and regulation of fusion proteins. (**A**) Although the parent proteins that constitute fusion proteins tend to be more highly expressed than average, the expression of fusion proteins tends to be low. Fusion protein expression is highly tissue specific and tends to follow the tissue distribution of the parent proteins. (**B**) 5′ translocation partners tend to have highly active promoters and 3′ partners have especially stable UTR regions, which suggests an optimization for increasing fusion transcript and protein levels. (**C**) An increasing number of reports demonstrate that cancer-associated fusions can also be present in healthy, non-diseased tissue. (**D**) The translation of fusion transcripts into fusion proteins is relatively rarely confirmed, which may be partially due to false positive hits from fusion transcript detection algorithms.

The principles that govern the expression and regulation of fusion proteins are currently insufficiently understood, but several pilot studies have begun to sketch an initial portrait of fusion protein regulation. A screen of 7424 putative fusion transcripts used RNA sequencing and mass spectrometry to confirm the expression of 175 fusion transcripts in 16 human tissues (84). The expression of fusion proteins was generally found to be low, and much more tissue specific than for other proteins (Figure 4A). A survey of transcription read-through fusions in prostate cancer found a correlation between parent gene expression and fusion transcript abundance: 5′ and 3′ genes with higher expression were more likely to produce observable fusion transcripts. Furthermore, the expression and tissue specificity of the fusion transcript correlated with expression patterns of the upstream parent gene (1). In accord, a more recent study of transcription read-through fusions in prostate cancer demonstrated that fusion transcript expression is similar to parental expression, and that fusion transcript levels respond similarly to androgen and anti-androgen treatment (114). By contrast, a study of *cis*-spliced fusions in prostate cancer found that only half of the fusions were significantly expressed relative to the parent genes (8). These results suggest that different mechanisms of fusion formation may influence the expression levels of the fusion transcripts and proteins.

Similarly, a computational review of translocated genes in hematological cancers confirmed that fusion proteins tend to be lowly expressed and tissue specific, but also reported that the constituent parents of fusion proteins are more highly expressed than average (47). In particular, 5′ translocation partners tended to have increased promoter activity and 3′ partners tended to have increased 3′ UTR stability. In general, one of the fusion parents (typically the 5′ gene) was found to predominantly contribute to the overexpression of the fusion protein instead of contributing structural features such as domains. Hence, 5′ partners appear to supply expression gains while 3′ supply functional protein segments and stability, which together could increase the expression of fusion proteins (Figure 4B). Given these trends, it remains unclear why, in practice, fusion transcript and protein expression levels are so low (115) (Figure 4C). A complicating aspect is the well-documented fact that false positive hits are a common occurrence in fusion transcript detection (116,117), which could artificially lower estimates of fusion protein expression by inflating the number of putative fusion transcripts.

Importantly, the expression of fusion proteins is not restricted to cancer tissue—the presence of fusion transcripts or proteins in normal cells has been known for years (39,84,118–121) (Figure 4D). For example, in the recent fusion screen of TCGA, 192 gene fusions were identified from 364 normal tissue samples (10). The function of fusions in healthy tissues is unclear, but has been suggested to increase the complexity of the proteome (115,119,122). After finding that translocation-induced fusion proteins seen in cancers were very rarely expressed in normal tissues, one study has suggested that there may be two, mostly non-overlapping sets of gene fusions—those associated with cancer and those found in normal tissue (84). However, the story behind the expression of fusion transcripts is likely much more complex—for instance, the recurrent *VTI1A-TCF7L2* fusion has been found in 42% samples of colorectal cancer samples, but also in 29% of normal colonic mucosa samples and, remarkably, in 25% of tested normal tissues from other organs (123). Further, the overactive production of certain apparently ‘normal’ fusions has been associated with cancer: for example, the constitutive expression of the *JAZF1-JJAZ1* fusion protein is pro-neoplastic in endometrial stromal sarcoma, but the same fusion protein is also found in benign tissues at lower levels (4,124). Similarly, the *SLC45A3-ELK4* fusion transcript is detected in both prostate cancer and benign prostate tissue, but is expressed at a higher level in the cancerous state (125). Further studies—especially global analyses that can capture the dominant trends of fusion protein expression across many (diseased and normal) human tissues—are urgently called for.

## DETECTING, PRIORITIZING AND ORGANIZING ONCOGENIC GENE FUSIONS

Historically, gene fusions have been associated with hematological cancers, partially because the complexity of the genomic changes in solid tumors confounded the available molecular cytogenetic methods (126). The advent of next-generation sequencing (NGS) technology, especially paired-end transcriptome sequencing (PE RNA-seq), and the development of complementary bioinformatics algorithms have revolutionized the detection of gene fusions and underscored the importance of fusions in solid tumors (34,127–129). These improved methods have discovered numerous novel gene fusions critical for cancer development (130). For example, bioinformatics approaches led to the discovery of the *TMPRSS2-ETS* gene fusion in prostate cancer (129) and the *EML4-ALK* fusion in non-small-cell lung cancer (66). The clinical importance of detecting *bona fide* gene fusions in tumor cells translates into a need for highly accurate and sensitive fusion detection and prioritization, while the rise in the number of gene fusion studies necessitates specialized databases and web services. Although gene fusion algorithms have been well-reviewed, prioritization algorithms and databasing work requires further dissemination.

### Detecting gene fusions in cancer

In recent years, there has been a remarkably concerted effort to develop algorithms and tools for identifying gene fusions from sequencing data. The first dedicated software, FusionSeq, was published in 2010 (131); by the end of 2012, 15 other tools had been released. At present, we find 30 different methods for identifying gene fusions (131–158) (Table 1), with the contenders for the most widely used packages being TopHat-Fusion (157) and deFuse (152). In addition, many other software packages, such as BreakDancer (159) and CREST (160), can call gene fusions in addition to other structural rearrangements. In the last 2 years, several additional fusion mapping tools have been released—FusionMetaCaller (161), JAFFA (133), IDP-fusion (132), TRUP (134), FusionCatcher and PRADA (136). A number of supporting tools, such as the Bioconductor package Chimera (162), offer utilities for organizing, analyzing and validating gene fusion lists reported by detection tools.

**Table 1.** Software packages, algorithms and tools for identifying gene fusions from sequencing data

Name	Notable features	URL	PMID	Year
**FusionMetaCaller**	An ensemble of the three fusion transcript detection algorithms (SOAPfuse, FusionCatcher and JAFFA) with the best performance on three synthetic and three real PE RNA-seq cancer data sets. R package.	http://tsenglab.biostat.pitt.edu/software.htm	26582927	2015
**INTEGRATE**	Combines WGS for structural variant detection with RNA-seq to detect expressed gene fusion transcripts. Emphasizes the minimization of false positive hit rate.	https://sourceforge.net/projects/integrate-fusion/	26556708	2015
**IDP-fusion**	Detects gene fusions, identifies junctions and quantifies fusion isoforms by integrating third-generation sequencing long reads and second-generation sequencing short reads.	http://www.healthcare.uiowa.edu/labs/au/IDP-fusion/	26040699	2015
**JAFFA**	Fusion transcript detection algorithm optimized for reads of 100 base pairs or greater. Uses a known transcriptome as an alignment reference instead of genome.	https://github.com/Oshlack/JAFFA/wiki	26019724	2015
**TRUP**	Detects fusion transcripts from PE RNA-seq data. Performs split read mapping and assembly of potential breakpoint regions. Filters include thresholds on repeat content and number of supporting reads.	https://github.com/ruping/TRUP	25650807	2015
**ChildDecode**	Detects several predefined pathognomonic gene fusions in childhood sarcomas from RNA-seq data. Operates on cloud-computing platform. Part of the FUSIONCloud commercial analytical platform.	http://www.fusiongenomics.com/onetest-products/	24517889	2014
**FusionCatcher**	Detects somatic fusion transcripts. Uses ensemble approach of four different methods and aligners to identify fusion junctions. Discordantly mapping reads are filtered on gene identity and positioning.	https://github.com/ndaniel/fusioncatcher	http://dx.doi.org/10.1101/011650	2014
**PRADA**	Uses dual-mapping strategy of aligning paired-end reads to a combined genome and transciptome reference to detect fusion transcripts. Outputs fusion frame classification, homology scores and other summary features.	http://sourceforge.net/projects/prada/	24695405	2014
**EBARDenovo**	Method for *de novo* assembly of short RNA-seq reads with a focus on detecting fusion transcripts. Optimized to handle confounding assembly errors. sequencing errors, repetitive sequences and amplicons.	http://ebardenovo.sourceforge.net/	23457040	2013
**FusionQ**	Detects gene fusions from PE RNA-seq data, reconstructs features of fusion transcripts and estimates their abundances. Uses a residual sequence extension method to extend short reads.	https://sites.google.com/site/fusionq1/home/	23768108	2013
**iFUSE**	Web-based visualization tool for structural variants that prioritizes breaks that are likely to be associated with gene fusions. Provides candidate transcript and protein sequences resulting from the identified gene fusions.	http://ifuse.erasmusmc.nl	23661695	2013
**SOAPFuse**	Identifies fusion transcripts through discordant PE reads and junction spanning reads. Features an improved algorithm for constructing the putative junction library and a relatively high computational efficiency.	http://soap.genomics.org.cn/soapfuse.html	23409703	2013
**SOAPfusion**	Part of the SOAP software for genome-wide detection of gene fusions from PE RNA-Seq data. Focuses on high sensitivity and low false discovery rates at low coverage.	http://soap.genomics.org.cn/SOAPfusion.html	24123671	2013
**Bellerophontes**	Identifies fusion transcripts from PE data. Selects from fusion candidates using a 'gene fusion model', and features splice site and abundance analyses that provide a more accurate set of junction reads.	http://eda.polito.it/bellerophontes/	22711792	2012
**BreakFusion**	Detects fusion transcripts using a targeted transcriptome assembly strategy. Introduces a single statistical chimeric score that summarizes the likelihood of a junction sequence containing true breakpoints.	http://bioinformatics.mdanderson.org/main/BreakFusion	22563071	2012
**elDorado**	Commercial software for identifying fusions from paired-end RNA seq reads. Filters on fusion structure and read support. Introduces the Transcriptome Viewer, a tool for visualizing gene fusions.	https://www.genomatix.de/online_help/help_eldorado/introduction.html	23036331	2012
**EricScript**	Detects fusion transcripts from PE data. Can create synthetic gene fusions with the EricScript simulator, and EricScript CalcStats can generate summary statistics for scoring fusion detection methods.	https://sites.google.com/site/bioericscript/	23093608	2012
**FusionAnalyser**	A graphical tool for detecting fusion transcripts from PE data that provides a user-friendly GUI and filtering system for non-programmers.	http://www.ilte-cml.org/FusionAnalyser/	22570408	2012
**FusionFinder**	Identifies gene fusion partners from either SE or PE RNA-seq data. Filters on features including read-through transcripts, homology and antisense information.	http://bioinformatics.childhealthresearch.org.au/software/fusionfinder/	22761941	2012
**LifeScope**	GUI-based splice and fusion detection from RNA-seq data method. Available from within the LifeScope software package.	https://www.thermofisher.com/uk/en/home/technical-resources/software-downloads/lifescope-genomic-analysis-software.html	22496636	2012
**nFuse**	Detects fusion transcripts and related chromosomal rearrangements from matched RNA-seq and whole genome shotgun sequencing data.	https://code.google.com/p/nfuse/	22745232	2012
**ChimeraScan**	Detects fusion transcripts from PE RNA-seq data. Automatically generates HTML reports to facilitate results analysis.	http://code.google.com/p/chimerascan/	21840877	2011
**Comrad**	Performs an integrated analysis of RNA-Seq and WGS data to detect genomic rearrangements and fusion transcripts. Handles low coverage genome data.	http://code.google.com/p/comrad/	21478487	2011
**deFuse**	Uses discordant paired end alignments to guide the split read analysis. Does not discard ambiguously mapping reads, but considers all possible alignments and fusion boundaries and resolve the most probable position.	http://sourceforge.net/apps/mediawiki/defuse/	21625565	2011
**FusionHunter**	Detects fusion transcripts from PE data. Can identify transcript fragments without known annotations. Filters on anchor length, read-through transcripts, junction reads and PCR artifacts.	http://bioen-compbio.bioen.illinois.edu/FusionHunter/	21546395	2011
**FusionMap**	Fusion gene detection from either SE or PE RNA-seq or gDNA-seq data. Focuses on improving the accuracy of mapping junction-spanning single reads.	http://www.omicsoft.com/fusionmap/	21593131	2011
**ShortFuse**	Fusion transcript detection from PE RNA-seq data. Focuses on accurately identifying fusion transcripts when many read pairs map ambiguously.	https://bitbucket.org/mckinsel/shortfuse	21330288	2011
**SnowShoes-FTD**	Fusion transcript detection from PE RNA-seq data. Includes prediction of genomic rearrangements, fusion protein sequence reconstruction and generation of fusion spanning sequence for PCR validation.	http://mayoresearch.mayo.edu/mayo/research/biostat/ stand-alone-packages.cfm	21622959	2011
**TopHat-Fusion**	A version of TopHat specialized for the detection of fusion transcripts. Implements a two stage process of aligning reads to genomic reference using altered version of TopHat, then a processing step to incorporate annotation and filter candidates.	http://tophat-fusion.sourceforge.net/	21835007	2011
**FusionSeq**	Fusion transcript detection from PE RNA-seq data. Considers annotated exons during mapping procedure, and reports read-through fusions in addition to other fusions. Variety of filters, including comparing fusion expression with general expression.	http://archive.gersteinlab.org/proj/rnaseq/fusionseq/	20964841	2010

GUI = graphical user interface, PCR = polymerase chain reaction, PE = paired-end, RNA-seq = RNA sequencing, SE = single end, WGS = whole genome sequencing.

The mechanisms, performance and features of different gene fusion detection algorithms have been well reviewed (130,163–166). Practical concerns—like the memory usage and computing time of detection algorithms—limit some tools (134), but this constraint is likely to decrease in importance as computing power continues to expand. Most recently, the performance and computational cost of 15 popular fusion detection algorithms was evaluated under a variety of experimental conditions, and a meta-caller algorithm that blended the three top performing methods to produce improved predictions was released as an R package (161). Meta-algorithms, or ensembles of different algorithms, often improve classification performance (167) and are likely to become more popular in fusion detection, especially since different fusion detection algorithms can be plagued by little predictive overlap (142,168). The difficulties of calling genuine gene fusions—including the complexity and instability of many cancer genomes, and technical errors in the sequencing or alignment procedure—are also well covered (116,117,130,169). In the rest of this section, we focus on methods for deciding which fusions are likely to be drivers and on gene fusion databases.

### Identifying driver gene fusions

Given the unprecedented sensitivity of gene fusion detection, and the repeated identification of fusion transcripts in normal cells, it is increasingly important to separate driver fusions from passenger mutations. Although many fusion detection tools encode their own filters in order to cut down on false positive calls (166), the criteria are most often based on read mapping quality and the presence of certain sequence features. Biological approaches that rank fusion candidates by some notion of functional importance are complementary and can offer a significant improvement in removing false positive calls.

The first integrative bioinformatics study with the goal of distinguishing ‘driver’ from ‘passenger’ fusions in high-throughput data took a gene-centric approach, ranking each gene by its similarity to ‘molecular concepts’ characteristic of cancer genes (49). These characteristics included specific functional annotations, pathway involvements protein interactions and domains. Interestingly, domain architectures and shared pathways were not nearly as indicative of cancer-related fusion genes as specific gene ontologies and the engagement of distinct interaction networks (e.g. fusion genes in acute lymphoblastic lymphoma tend to frequently interact with *GATA3*) (49). Notably, while point mutated cancer genes tended to be involved in DNA repair and cell cycle checkpoints, driver fusions tended to include genes with signal transduction and transcription activation functions. Further, by analyzing high-throughput copy number genomic data, recurrent gene fusion events were found to be associated with consistent, specific patterns of copy number alteration. These trends were used to design an algorithm for ranking genes by their ability to form driver fusions.

Prioritization of gene fusions using characteristics from only one gene is necessarily incomplete, because gene fusions generally involve two partner genes. Wu *et al*. addressed this concern using the concept of network centrality (170). They observed that in most known cancer fusion gene pairs, at least one of the fusion partners acts as a hub (i.e. has many interaction partners) in a gene interaction network (where genes are nodes and edges indicate a regulatory or protein–protein interaction). Many fusions were found to be formed from two hubs, possibly because the central positioning of hub-like genes confers a large radius of influence, maximizing the deregulation of other genes and pathways if they are fused or disrupted. A network centrality based classifier was developed for scoring fusions, which showed superior performance compared to both the above method (49) and a simpler gene-based model that selects drivers based on whether the fusion includes a cancer-associated gene.

Oncofuse (95) innovated the use of fusion sequences, instead of gene qualities, to identify driver fusions. First, a set of 24 features of fusion transcripts was built up, including functional profiles, tissue-specific expression, replication timing of the gene-containing locus, interaction partners, interaction partner expression, 3′ UTR length and domains. Notably, both retained and lost features were included in the dataset, e.g. the domains that were both lost and retained as a result of the fusion breakpoint position. A Naïve Bayes classifier was trained on these feature sets, which contained both positive data (known oncogenic fusions) and negative data (fusion genes and read-through transcripts found in normal cells). Functional profile information provided the largest information gain for classification—molecular functions related to transcription factors, kinases and histone modification were highly enriched in driver fusions. This echoes previous results (49). Expression and replication features were most important for 5′ partners, which also resonates with other literature (1,47). Interestingly, certain lost features, like protein interaction interfaces, were more informative than the retained features.

The most recent method for nominating fusion drivers is the Pegasus pipeline (86), which emphasizes transcript sequence reconstruction and domain annotation. Pegasus extends the Oncofuse domain analysis by considering reading frame conservation and all possible isoforms. Specifically, Pegasus reconstructs the fusion transcript sequence for each gene fusion candidate, annotates breakpoints as occurring in the CDS, introns or UTRs, and assesses reading frames. Lost and retained domains of the 5′ and 3′ partners are determined, and certain domain features (e.g. oncogenic domains) are annotated. A gradient tree boosting algorithm is trained on positive driver fusions from ChimerDB 2.0 (171) and on a complex negative dataset (composed of passenger fusions, read-through transcripts in normal tissue, etc.). In-frame transcripts were found to by far be the most distinguishing feature of driver fusions. Other important factors included breakpoints in the CDS and domains from known oncogenes (or domains interacting with known oncogenes). Pegasus performed well on the curated validation set and on real RNA-seq data, and outperformed Oncofuse in several tests. It is probable that the identification of driver fusions, either with these existing tools or with new methods, will play an increasingly important role in cancer research as the number and size of fusion-detecting studies continues to expand.

### Curating knowledge on oncogenic gene fusions

The rapid increase of gene fusion data requires significant organizational effort, and at present almost a dozen databases of oncogenic fusion genes exist (Table 2). Some of the earliest efforts to catalog gene fusions, such as the Atlas of Genetics and Cytogenetics in Oncology and Haematology (172), arose before the advent of deep sequencing of the transcriptome. Most currently available databases leverage sequence technology advances but vary significantly in their methodology, focuses and sizes—for example, the ∼29 000 fusion transcripts in ChiTaRS result mainly from bioinformatics analyses of public databases, while the ∼2600 chromosome rearrangements in dbCRID are manually curated from the literature. Here, we outline the progress made by recent gene fusion databases. We only focus on databases that concentrate on gene fusions, but related resources such as the database of genomic variants (173) or the DECIPHER database of chromosome imbalances and phenotypes (174) also include gene fusions.

**Table 2.** Databases of gene fusions

Database name	Description	Data sources	URL	PMID	Database size (in current release or as of October 2015)	First published and current database release
**Mitelman**	Relates gene fusions and other chromosomal aberrations to tumor characteristics, based either on individual cases or associations.	Manual literature curation.	http://cgap.nci.nih.gov/Chromosomes/Mitelman	17361217 (review)	10 026 gene fusions; 65 975 patient cases	1994–2015. Current release: August 2014
**COSMIC Curated Fusions**	Catalog of translocations and fusions between gene pairs supplemented with extensive clinical data.	Manual literature curation.	http://cancer.sanger.ac.uk/cosmic/help/fusion/overview	25355519 (full 2015 COSMIC db)	10 534 gene fusions	2004–2015. Current release: v70 (2014)
**FARE-CAFÉ**	Database of functional and regulatory elements in gene fusion events related to cancer.	Integration of diverse data sets, including fusion events, molecular and regulatory features.	http://ppi.bioinfo.asia.edu.tw/FARE-CAFE.	26384373	1587 gene fusions	2015
**TCGA Fusion Gene Data Portal**	Repository for the results of the landscape of cancer-associated fusion study carried out using the PRADA pipeline.	Integrated analysis of paired-end RNA sequencing and DNA copy number data from TCGA.	http://54.84.12.177/PanCanFusV2/	25500544	7887 fusion transcripts	2015. Current release: December, 2014.
**FusionCancer**	Fusion gene database derived exclusively from cancer RNA-seq data.	Compiled from 591 recently published RNA-seq datasets covering 15 kinds of human cancer.	http://donglab.ecnu.edu.cn/databases/FusionCancer/	26215638	11 839 gene fusions	2015
**ChiTaRS**	Catalogue of fusion transcripts in humans, mice, fruit flies, zebrafishes, cows, rats, pig and yeast.	Bioinformatic analysis of ESTs and mRNAS from GenBank.	http://chitars.bioinfo.cnio.es/	25414346 (2.1); 23143107	29 159 fusion transcripts	2013. Current release: ChiTaRS 2.1 (2014)
**dbCRID**	Curated database of human chromosomal rearrangements, associated diseases and clinical symptoms.	Manual literature curation.	http://dbcrid.biolead.org/	21051346	2643 chromosome rearrangements	2011. Current release: v 0.9 (2010)
**ConjoinG**	Database of conjoined genes (transcription read-through fusions).	Manual literature curation and bioinformatic analysis of EST and mRNA sequences from GenBank.	http://metasystems.riken.jp/conjoing/	20967262	800 conjoined genes from 1542 parent genes	2010
**HYBRIDdb**	Database of hybrid genes in the human genome.	Analysis of mRNA, EST, cDNA and genomic DNA sequences in the INSDC resource.	http://www.primate.or.kr/hybriddb/	17519042	3404 gene fusions	2007
**TICdb**	Finely mapped translocation breakpoints in cancer.	Manual literature curation and analysis of public databases (Mitelman, GenBank).	http://www.unav.es/genetica/TICdb/	17257420	1374 fusion sequences from 431 different genes	2007. Current release: release 3.3 (2013)
**ChimerDB**	Knowledgebase of fusion transcripts across multiple species, as well as information on cancer breakpoints.	Bioinformatics analysis of Sanger CGP, OMIM, PubMed and the Mitelman's database and transcript sequences in GenBank.	http://biome.ewha.ac.kr:8080/FusionGene/	19906715 (2.0); 16381848	2699 fusion transcripts	2006. Current release: ChimerDB 2.0 (2010)
**DACRO**	Database of all published chromosomal rearrangements that are associated with an abnormal phenotype.	Online searches of PubMed, Scopus and OMIM.	https://www1.hgu.mrc.ac.uk/Softdata/Translocation/	Unpublished	965 translocations	NA

Databases are annotated with source data types, URLs, estimates of database content and size and first and current releases. EST = expressed sequence tag, INSDC = International Nucleotide Sequence Database Collaboration, OMIM = Online Mendelian Inheritance in Man, Sanger CGP = Sanger Cancer Genome Project, TCGA = The Cancer Genome Atlas.

Initial efforts to catalog gene fusions included (early versions of) the Mitelman database, COSMIC (175), ChimerDB (171,176), TICdb (177) and HYBRIDdb (178). The Mitelman database of chromosomal aberrations and gene fusions began as early as 1994, appearing in print and on CD-ROM, and has grown into one of the most popular current resources on gene fusions (179). This heavily curated database of fusions is supplemented with clinical association information, like karyotype abnormalities associated with a particular tumor type or patient prognosis. The database is searchable by a wide variety of fields, such as patient age, publication authors, gene, tumor histology, tissue type, mutation recurrence, associated clinical features and cancer types. Similarly, the COSMIC catalog of somatic mutations in cancer offers an extensive curated collection of oncogenic gene fusions. Initially published in 2004 (175), the COSMIC database has also grown to significant size and scope (180). COSMIC's fusion information is manually curated from the literature (though currently only for solid tumors) and incorporates information on inferred breakpoints, included exons of the 5′ and 3′ partners, and mutation frequency. Extensive clinical data are also integrated, such as patient details, tumor (stage/drug response) and sample features (histology, source) and tissue-specific fusion mutation frequencies.

Another relatively early fusion resource, ChimerDB, was initially published in 2006 (171) and now houses 2700 fusion transcripts (176), which were identified via bioinformatics analysis of GenBank, the NCBI short read archive, Sanger CGP, OMIM, Mitelman's database and PubMed. The ChimerDB computational pipeline involves aligning fusion sequences to the reference human genome and classifying gene fusions into confidence classes based on the transcript fusion boundary matching exon boundaries. The service offers support for detailed searches (e.g. by gene, chromosomal band and tissue), filtering by alignment (e.g. intact exons at breakpoint) and an alignment viewer. Interestingly, the ChimerDB study reported that the overlap between Sanger CGP, OMIM, Mitelman and their PubMed gene fusion list was relatively small, with almost 60% of fusion pairs found in only one resource, indicating a strong need to integrate fusion datasets. TICdb, a resource of translocation breakpoints in cancer published the year after ChimerDB, is a highly curated database that sought to address the lack of molecular information on gene fusions in the Mitelman database (177). It was also the first repository to map translocation breakpoints onto the reference genome and provide fusion boundary sequences. TICdb was built by extracting 298 genes involved in reciprocal translocations from the 2006 version of the Mitelman database and searching PubMed and Genbank to find translocation junction sequences. Another early effort by HYBRIDdb (178) identified 3400 gene fusions from a bioinformatics analysis of mRNA, EST, cDNA and transcript sequences in the NCBI database. Unfortunately no longer functional, the HYBRIDdb resource mapped fusion transcripts and junctions, classified fusions into translocation and transcription-mediated categories and integrated information on splicing sites, domains and associated pathologies and affected tissues.

The ConjoinG (181) and dbCRID (182) databases represented the next wave of fusion databases. The development of the ConjoingG database of ‘conjoined genes’, or transcription-induced fusion genes, sought to address the lack of uniformity in annotating conjoined genes in the UCSC, GenBank, Ensembl and Vega databases. ConjoinG computationally identified and curated 800 read-through transcripts that were supported by one or more mRNA or EST sequence in NCBI. Representative conjoined genes were selected and subjected to validation by RT-PCR and sequencing, and the evolutionary conservation and splicing patterns of these fusion events were analyzed. On the other hand, the dbCRID database of chromosomal rearrangements in human diseases focused on documenting chromosomal rearrangements in both tumor and non-tumor diseases, covering several types of chromosomal rearrangements.

The most recent gene fusion databases include ChiTaRS 2.1 (183,184), FusionCancer (168), the TCGA Fusion Portal (10) and FARE-CAFE (88). The ChiTaRS 2.1 database of fusion transcripts and RNA-seq data (184) is the largest fusion databases and one of the few containing non-human fusion data. It catalogs over 29 000 fusion transcripts, largely from humans, mice and fruit flies. The fusion transcripts were identified via bioinformatics analysis of ESTs and mRNAs from several databases, and some fusion transcripts also have associated expression and tissue specificity data.

The FusionCancer database is a unique gene fusion repository derived solely from the analysis of raw cancer RNA-seq data (168). To create FusionCancer, 591 recent RNA-seq datasets from 15 cancer types were compiled and gene fusions identified using several fusion detection packages. Interestingly, though ∼12 000 gene fusions were identified with at least one software, only 137 fusions were identified by all four. FusionCancer implements information from COSMIC and ChimerDB, and includes breakpoint location, recurrence rate and fusion sequences. A similar methodology underlies the TCGA Fusion Portal—using stringent bioinformatic criteria, Yoshihara and coworkers identified over 8600 distinct fusion transcripts from data on 13 different cancer types in the Cancer Genome Atlas (TCGA), many involving genes not previously known to form fusions (10). These results have illuminated the roles of many gene fusions (see **Section II**) and have been integrated into other resources, such as the Mitelman database. Finally, the most recently released fusion database is FARE-CAFE (88), a resource of functional and regulatory elements in fusions. This highly integrated database aims to summarize how fusions affect a variety of molecular components and activities, including Pfam domains, domain–domain interactions, protein–protein interactions and transcription factor functions.

It is clear that our understanding of the number of gene fusions and their potential functions is far from being complete. Notably, many fusion databases have very limited overlap between the fusions they document. This is certainly partially due to methodological differences in detection and filtering. However, considering the rate at which novel oncogenic fusions are still being discovered (10,12,57), it is likely that we have still only detected a small fraction of existing fusions, and future research will continue to gradually improve coverage. Furthermore, current databases reflect the fact that we have little knowledge of certain classes of fusion, such as fusions involving genes encoding long non-coding RNAs, despite their documented central role in cancer (105–107). Finally, although many studies and databases understandably focus on oncogenic fusions, exploring certain poorly understood aspects of fusions which are perhaps not directly related to cancer is likely to synergistically improve our understanding of cancer-related fusions. Such topics include the role of fusions in healthy tissues and non-human organisms, the functions of singleton fusions, and the extent to which ‘known’ fusions are false positive results. Indeed, our intuition of what constitutes a ‘functional’ fusion event (e.g. being recurrent, in-frame and only present in diseased tissues) is likely to transform as more information is acquired on these topics. The substantial number of unknown facets of gene fusion functionality presents truly exciting opportunities for future discovery.

## CONCLUSIONS AND FUTURE DIRECTIONS

The computational study of fusion genes, transcripts and proteins is still in its infancy. The improved detection and functional characterization of these frequently oncogenic mutations will continue to play an important role in elucidating cancer processes across diverse tumor types. The recent successes in the development of drugs against mutated kinases and chromatin modifying proteins (59,82,185,186), and novel methods of therapeutically downregulating proteins in general (187,188), suggest that fusion transcripts and proteins are likely to be promising targets for the next generation of therapeutic agents against cancer, and data-intensive studies of gene fusions have the key role of directing these future avenues of medical research.
